# Quantitative assessment of the universal thermopower in the Hubbard model

**DOI:** 10.1038/s41467-023-42772-8

**Published:** 2023-11-03

**Authors:** Wen O. Wang, Jixun K. Ding, Edwin W. Huang, Brian Moritz, Thomas P. Devereaux

**Affiliations:** 1 Department of Applied Physics, Stanford University, Stanford, CA 94305 USA; 2 Stanford Institute for Materials and Energy Sciences, SLAC National Accelerator Laboratory, 2575 Sand Hill Road, Menlo Park, CA 94025 USA; 3 Department of Physics and Institute of Condensed Matter Theory, University of Illinois at Urbana-Champaign, Urbana, IL 61801 USA; 4 Department of Physics and Astronomy, University of Notre Dame, Notre Dame, IN 46556 USA; 5 Stavropoulos Center for Complex Quantum Matter, University of Notre Dame, Notre Dame, IN 46556 USA; 6 Department of Materials Science and Engineering, Stanford University, Stanford, CA 94305 USA; 7 Geballe Laboratory for Advanced Materials, Stanford University, Stanford, CA 94305 USA

**Keywords:** Electronic properties and materials, Quantum fluids and solids, Superconducting properties and materials, Thermodynamics

## Abstract

As primarily an electronic observable, the room-temperature thermopower *S* in cuprates provides possibilities for a quantitative assessment of the Hubbard model. Using determinant quantum Monte Carlo, we demonstrate agreement between Hubbard model calculations and experimentally measured room-temperature *S* across multiple cuprate families, both qualitatively in terms of the doping dependence and quantitatively in terms of magnitude. We observe an upturn in *S* with decreasing temperatures, which possesses a slope comparable to that observed experimentally in cuprates. From our calculations, the doping at which *S* changes sign occurs in close proximity to a vanishing temperature dependence of the chemical potential at fixed density. Our results emphasize the importance of interaction effects in the systematic assessment of the thermopower *S* in cuprates.

## Abstract (web summary)

High-temperature behaviour of thermopower is special in cuprates, allowing for theory-experiment comparisons. Wang et al. use quantum Monte Carlo to compute high temperature thermopower in the Hubbard model, demonstrating qualitative and quantitative agreement with experiments across multiple cuprate families.

## Introduction

The Hubbard model, despite decades worth of study, remains enigmatic as a model to describe strongly correlated systems. Due to the fermion sign problem and exponential complexity, only one-dimensional systems have lent themselves to error-free estimations of ground states and their properties. Recently, angle-resolved photoemission studies have demonstrated that a one-dimensional Hubbard-extended Holstein model can quantitatively reproduce spectra near the Fermi energy^1–3^. In two dimensions, the community lacks exact results in the thermodynamic limit; nevertheless, many of the extracted properties from simulations of the Hubbard model bear a close resemblance to observables measured in experiments, particularly those performed on high-temperature superconducting cuprates. These properties include the appearance of antiferromagnetism near half-filling, stripes, and strange metal behavior^4–6^. However, quantitative assessments have remained out of reach, particularly regarding transport properties, where multi-particle correlation functions (calculations involving the full Kubo formalism) are computationally intensive, or one must rely on single-particle quantities (i.e., Boltzmann formalism), which can be conceptually problematic for strong interactions.

In principle, the high-temperature behavior of the thermopower (thermoelectric power, or Seebeck coefficient) *S* offers the possibility to directly test the Hubbard model against experiments in strongly correlated materials like the cuprates. Above the Debye temperature, phonons are essentially elastic scatterers of electrons and one might expect thermal relaxation to come overwhelmingly from inelastic scattering off of other electrons. Moreover, room-temperature measurements afford direct contact with determinant quantum Monte Carlo (DQMC)^7,8^ simulations, which are limited by the fermion sign problem to temperatures above roughly *J*/2 (half of the spin-exchange energy). Thus, one can address directly an essential question—can the Hubbard model give both qualitative and quantitative agreement with the observed thermopower in cuprates at high temperatures?

Systematic studies of the room-temperature thermopower across a wide variety of cuprates^9–17^ show that the thermopower falls roughly on a universal curve over a broad range of hole doping *p*, with a more-or-less universal sign change near optimal doping. This sign change has been interpreted as evidence for a Lifshitz transition^18–20^; however, this implies that the doping associated with the sign change depends on material specifics and the detailed shapes of Fermi surfaces, which is hard to reconcile with the observed universality. An alternative interpretation of the sign change appeals to the atomic limit^21–27^; however, the atomic limit requires extremely strong interactions and a very high temperature *T* compared to the bandwidth, neither of which is satisfied in cuprates at room temperature. The thermopower *S* also has been approximated by the entropy per density, defined through the Kelvin formula *S*_Kelvin_ = (∂*s*/∂*n*)_*T*_/e^*^^28^, where charge e^*^ = − *e* for electrons. *S*_Kelvin_ is believed to be an accurate proxy for the thermopower *S*, since it accounts for the full effects of interactions, while bypassing the difficulties in exactly calculating the Kubo formula^25,28–30^. However, a direct comparison between *S* and *S*_Kelvin_ is required before drawing any conclusions based on these assumptions.

Here, we calculate the thermopower *S* based on the many-body Kubo formula, as well as the Kelvin formula *S*_Kelvin_, for the *t*-${t}^{{\prime} }$-*U* Hubbard model. We employ numerically exact DQMC and maximum entropy analytic continuation (MaxEnt)^31,32^ to obtain the DC transport coefficients that specifically enter the evaluation of *S*. Our results show that the Hubbard model can quantitatively capture the magnitudes and the general patterns of *S* that have been observed in cuprate experiments.

## Results

**Fig. 1:**
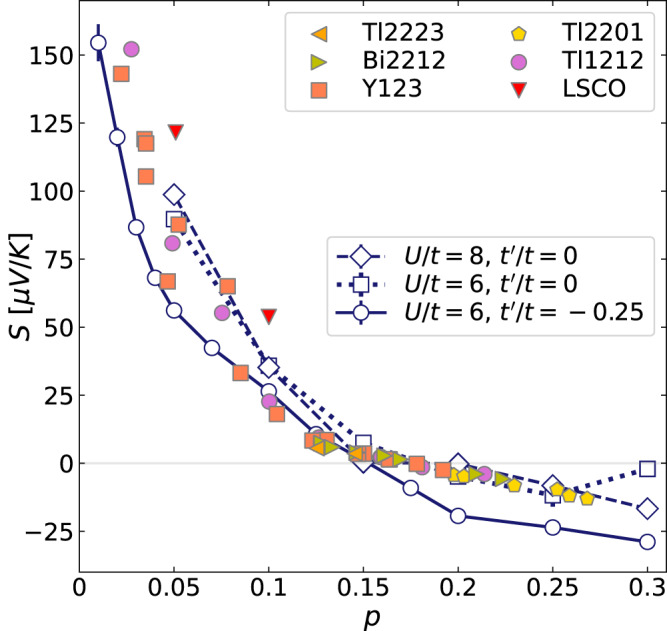
Comparison of simulated and experimental thermopower. Thermopower *S* as a function of doping *p* from DQMC simulations (empty markers connected by lines), compared with doping dependence of *S* for various cuprates at *T* = 290 *K* (solid scattered markers, data from refs. ^9,11^). For *U*/*t* = 8 and ${t}^{{\prime} }/t=0$, the temperature is *k*_*B*_*T* = *t*/3.5. For *U*/*t* = 6, the temperature is *k*_*B*_*T* = *t*/4 for both ${t}^{{\prime} }/t=0$ and ${t}^{{\prime} }/t=-0.25$.

The doping dependence of thermopower *S* from the Hubbard model is shown in Fig. 1 for three different sets of parameters at their lowest achievable temperatures, overlaid with experimental data from several families of cuprates. It is important to note that in the process of converting our results to real units based on universal physical quantities *k*_*B*_ and *e*, there are no adjustable parameters: *S* is a ratio, so the standard units of *t* (or *U*) in the Hubbard model factor out. The most striking observation is the surprisingly good agreement between our results and the room-temperature thermopower in cuprates, in both qualitative trend and quantitative magnitudes. Both the simulation and experimental data show a sign change roughly at *p* ~ 0.15. In both cases, *S*—a quantity proportional to the electronic resistivity—increases dramatically in the low doping regime, as the system approaches a Mott insulator. The simulation shows moderate *U* and ${t}^{{\prime} }$ dependence, without significantly affecting agreement with experiments. The moderate parameter dependence is consistent with the observed approximate universality of the doping dependence of the room-temperature *S* for different cuprates, which may have varying effective *U* and ${t}^{{\prime} }$.

**Fig. 2:**
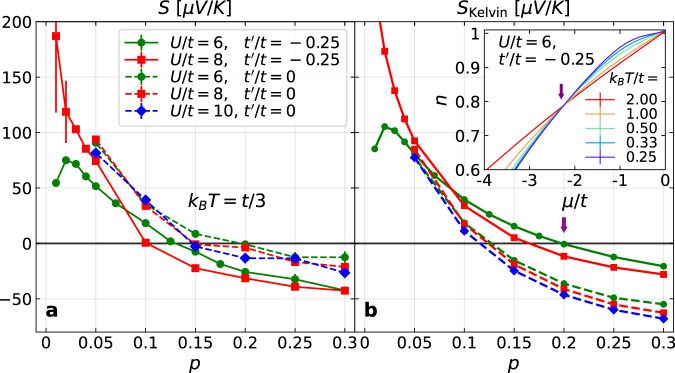
Doping dependence and sign change of *S* and *S*_Kelvin_. Thermopower *S* (**a**) and the Kelvin formula for the thermopower *S*_Kelvin_ (**b**) as a function of doping *p* for the Hubbard model with different *U* and ${t}^{{\prime} }$, all at the same temperature *k*_*B*_*T* = *t*/3. Inset of (**b**): density *n*, measured using DQMC, as a function of the chemical potential *μ* for *U*/*t* = 6, and ${t}^{{\prime} }/t=-0.25$ at different temperatures *T*. The arrows in (**b**) and its inset indicate the correspondence between the sign change of *S*_Kelvin_ and the vanishing of the temperature dependence of *μ* at fixed density.

For weakly interacting electrons, *S* is expected to change sign around the Lifshitz transition. The sign change in our model with strong interactions, which occurs at *p* ~ 0.15 for ${t}^{{\prime} }/t=-0.25$, is much lower than the Lifshitz transition, which occurs at *p* ~ 0.26 for the same parameters, nor is it associated with the atomic limit (see Supplementary Note 3 and Supplementary Note 5 for details). Therefore, we seek a deeper understanding from *S*_Kelvin_ = − (∂*s*/∂*n*)_*T*_/*e*, entropy variation per density variation at a fixed temperature, or equivalently, by the Maxwell relation, (∂*μ*/∂*T*)_*n*_/*e*, chemical potential variation per temperature variation at fixed density (see Supplementary Note 4). In Fig. 2, we compare the doping dependence of *S* and *S*_Kelvin_. Despite differences in exact values, the sign change of *S*, as shown in Fig. 2a, is closely associated with that of *S*_Kelvin_, as shown in Fig. 2b. The sign change of *S*_Kelvin_ occurs when the temperature dependence of the chemical potential *μ* vanishes at fixed density—an “isosbestic” point, as exemplified in the inset of Fig. 2b, and highlighted by the arrows.

The doping dependence of *S* and *S*_Kelvin_ are also qualitatively similar, and *U* generally affects both *S* and *S*_Kelvin_ in a similar manner, moderately reducing the doping at which each changes sign as *U* increases. However, ${t}^{{\prime} }$ has more significant and opposite effects on *S* and *S*_Kelvin_. Comparing Fig. 2a and b shows us that even though *S*_Kelvin_, a thermodynamic quantity, differs from *S*, since it does not reflect the dynamics captured by transport^33^, *S*_Kelvin_ still reflects the most important effects from the Hubbard interaction, showing a doping dependence and sign change similar to *S*.

**Fig. 3:**
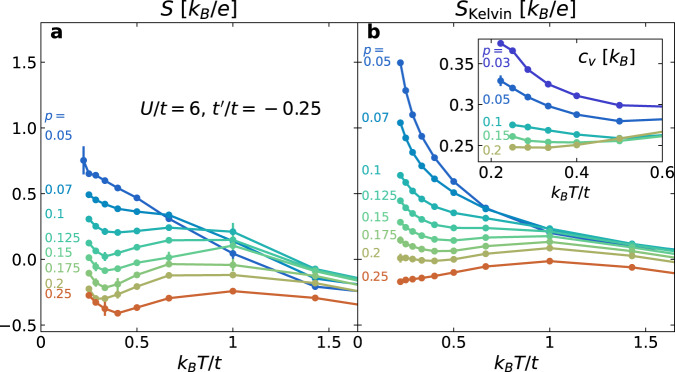
Temperature dependence of *S* and *S*_Kelvin_. Thermopower *S* (**a**), and the Kelvin formula for the thermopower *S*_Kelvin_ (**b**), as a function of temperature *T*, at different doping levels *p*, for *U*/*t* = 6, and ${t}^{{\prime} }/t=-0.25$. Inset of **b** shows the specific heat *c*_*v*_ measured using DQMC as a function of temperature for different doping levels.

We now examine the temperature dependence of *S* and *S*_Kelvin_, using *U*/*t* = 6 and ${t}^{{\prime} }/t=-0.25$, shown in Fig. 3, as a representative example. The temperature dependence of *S* in Fig. 3a and *S*_Kelvin_ in Fig. 3b are qualitatively similar. As temperature decreases from high temperatures, *S* and *S*_Kelvin_ first increase, following the atomic limit ($t,{t}^{{\prime} }\ll {k}_{B}T,U$, see Supplementary Note 5). As temperature decreases further and passes the scale *t*/*k*_*B*_, their behaviors deviate from the atomic limit. At low doping (*p* ≲ 0.07), *S* and *S*_Kelvin_ monotonically increase, but at higher doping levels, they first decrease before increasing again down to the lowest temperature, with a dip appearing in between.

We find the dip and the low-temperature increase in both *S* and *S*_Kelvin_ particularly interesting, since this upturn commonly appears in cuprates^9–11,13,16^, and cannot be understood in either the atomic or weakly interacting limits. To understand its origin, we consider the relationship between *S*_Kelvin_ and the specific heat *c*_*v*_ using the Maxwell relation $-e{(\partial {S}_{{{{{{{{\rm{Kelvin}}}}}}}}}/\partial T)}_{p}=-{(\partial {c}_{v}/\partial p)}_{T}/T$, where, by definition, *S*_Kelvin_ = (∂*s*/∂*p*)_*T*_/*e* and *c*_*v*_ = *T*(∂*s*/∂*T*)_*p*_. Specific heat *c*_*v*_ results, also for *U*/*t* = 6 and ${t}^{{\prime} }/t=-0.25$, are shown in the inset of Fig. 3b. Near half-filling and for temperatures below the spin-exchange energy *J* (=4*t*^2^/*U* to leading order), *c*_*v*_ starts to increase with decreasing temperatures, which is believed to be associated with spin fluctuations^34–36^, and *c*_*v*_ drops with increasing doping. Correspondingly, *S*_Kelvin_ at fixed doping increases with decreasing temperatures, leading to a low-temperature upturn. As the upturn is a common feature shared by *S* and *S*_Kelvin_, it is reasonable to believe that the origin should be the same.

The low-temperature slope of the thermopower can be compared with experiments. The negative slopes quoted in ref. ^11^ for Bi_2_Sr_2_CaCu_2_O_8+*δ*_ and Tl_2_Ba_2_CuO_6+*δ*_ range roughly from −0.05 to −0.02 *μV*/*K*^2^. Assuming *t*/*k*_*B*_ ~ 4000 *K*, this range corresponds to $\left[-2.3,-0.9\right]\,{k}_{B}^{2}/(te)$ in our model. We estimate the slope in our model by taking the finite difference between temperatures *k*_*B*_*T* = *t*/4 and *t*/3.5 in Fig. 3a and b. For doping between *p* = 0.1 and 0.2, the calculated slope ranges between $\left[-2.1,-1.5\right]\,{k}_{B}^{2}/(te)$ for *S*, and $\left[-1.8,-0.2\right]\,{k}_{B}^{2}/(te)$ for *S*_Kelvin_. Even though systematic and statistical errors in *S* introduce uncertainties to this slope estimate, the ranges are roughly comparable between simulated *S*, *S*_Kelvin_, and experimental values.

**Fig. 4:**
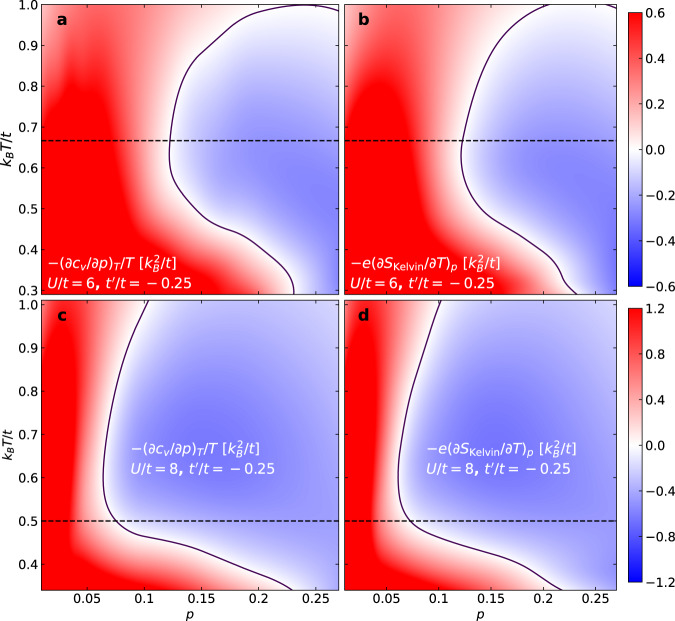
Analysis using *c*_*v*_ and *S*_Kelvin_. Color density plots of −∂^2^*s*/(∂*p*∂*T*) calculated from doping derivative of specific heat [$-{(\partial {c}_{v}/\partial p)}_{T}/T$, (**a**, **c**)] and temperature derivative of *S*_Kelvin_ [$-e{(\partial {S}_{{{{{{{{\rm{Kelvin}}}}}}}}}/\partial T)}_{p}$, (**b**, **d**)], for interaction strengths *U*/*t* = 6 (**a**, **b**) and *U*/*t* = 8 (**c**, **d**), both with ${t}^{{\prime} }/t=-0.25$. A cubic-spline fit was applied to curves of *c*_*v*_ versus *p* and *S*_Kelvin_ versus *T*, with corresponding derivatives obtained from the fits. The derivatives −∂^2^*s*/(∂*p*∂*T*) were interpolated (cubic) onto the two-dimensional (*p*, *T*) plane. Horizontal dashed lines mark the leading-order approximation for the spin-exchange energy *J* = 4*t*^2^/*U*, and solid lines mark the contour where −∂^2^*s*/(∂*p*∂*T*) = 0.

For a detailed verification and analysis of the relationship between *S*_Kelvin_ and *c*_*v*_, we calculate −∂^2^*s*/(∂*p*∂*T*) from derivatives of independently measured *S*_Kelvin_ and *c*_*v*_, for both *U*/*t* = 6 and *U*/*t* = 8 with ${t}^{{\prime} }/t=-0.25$, as shown in Fig. 4. Results from the two methods are consistent, up to minor discrepancies such as taking derivatives from discrete data points. At any point along the contour ∂^2^*s*/(∂*p*∂*T*) = 0 (black solid lines), either a peak or a dip will occur in *S*_Kelvin_ as a function of *T*. We observe that a peak appears at temperatures above *J*/*k*_*B*_ (dashed horizontal line) and a dip appears at temperatures below *J*/*k*_*B*_. Note that *T* ~ *J*/*k*_*B*_ corresponds roughly to the crossover between a peak or dip in *S*_Kelvin_ for both *U*/*t* = 6 and *U*/*t* = 8 (c.f. Supplementary Fig. 6), supporting our idea that the non-monotonic temperature dependence of both *S*_Kelvin_ and *S* should be associated with effects of spin exchange.

## Discussion

In summary, we calculated the thermopower *S* and the Kelvin formula *S*_Kelvin_ in the Hubbard model. *S* shows qualitative and quantitative agreement with the universal curve of the room-temperature *S* in cuprates, with a sign change corresponding to an “isosbestic” point in *n* versus *μ*. *S* and *S*_Kelvin_ show qualitatively similar doping dependence, and the doping at which *S* changes sign corresponds well to that of *S*_Kelvin_. As a function of temperature, we observe a low-temperature upturn in *S* and *S*_Kelvin_ with a slope quantitatively comparable with the corresponding linear increase in cuprates, and we provide evidence supporting their association with the scale of *J*. With this general agreement, we demonstrate that major features in the universal behavior of *S* in cuprates can be replicated through a quantitative assessment of *S* in the Hubbard model. The observation that *S*_Kelvin_ captures qualitative features of *S* enables us to understand the experimental thermopower results from the perspective of entropy variation with density.

We emphasize the significance of such a high level of agreement between simulations and experiments for thermopower. Transport properties can be sensitive to numerous factors, which may be different between cuprates and the *t*-${t}^{{\prime} }$-*U* Hubbard model. The combination of the model’s simple form and capability to reproduce universal features suggests the dominance of interaction effects in the origin of the systematic behavior in the cuprates. Our observations highlight the importance of pursuing high-accuracy simulations accounting for the full effect of interactions in making progress at understanding these enigmatic materials.

## Methods

We investigate the two-dimensional single-band *t*-${t}^{{\prime} }$-*U* Hubbard model with spin *S* = 1/2 on a square lattice using DQMC^7,8^. The Hamiltonian is

1 $$H=	 -t\mathop{\sum}\limits_{\langle lm\rangle,\sigma }\left({c}_{l,\sigma }^{{{{\dagger}}} }{c}_{m,\sigma }+{{{{{{{\rm{h.c.}}}}}}}}\right)\\ 	 -{t}^{{\prime} }\mathop{\sum}\limits_{\langle \langle lm\rangle \rangle,\sigma }\left({c}_{l,\sigma }^{{{{\dagger}}} }{c}_{m,\sigma }+{{{{{{{\rm{h.c.}}}}}}}}\right)\\ 	+U\mathop{\sum}\limits_{l}\left({n}_{l,\uparrow }-\frac{1}{2}\right)\left({n}_{l,\downarrow }-\frac{1}{2}\right),$$

where *t* (${t}^{{\prime} }$) is the nearest-neighbor (next-nearest-neighbor) hopping, *U* is the on-site Coulomb interaction, ${{c}}_{l,{\sigma }}^{{{{\dagger}}} }({{c}}_{l,{\sigma }})$ is the creation (annihilation) operator for an electron at site *l* with spin *σ*, and ${{n}}_{l,{\sigma }}\equiv {{c}}_{l,{\sigma }}^{{{{\dagger}}} }{{c}}_{l,{\sigma }}$ is the number operator at site *l* with spin *σ*.

The Kelvin formula for the thermopower *S*_Kelvin_ can be calculated using DQMC through

2 $${S}_{{{{{{{{\rm{Kelvin}}}}}}}}}=-\frac{\langle (H-\mu N)N\rangle -\langle H-\mu N\rangle \langle N\rangle }{eT(\langle NN\rangle -\langle N\rangle \langle N\rangle )},$$

where *N* = ∑_*l*_(*n*_*l*,*↑*_ + *n*_*l*,*↓*_) is the total electron number operator, and *μ* is the chemical potential.

From the Hamiltonian in Eq. (1), the particle current **J** and the energy current **J**_*E*_ are obtained as^37,38^

3 $${{{{{{{\bf{J}}}}}}}}=	 \frac{t}{2}\mathop{\sum}\limits_{l,{{{{{{{\boldsymbol{\delta }}}}}}}}\in {{{{{{{\rm{NN}}}}}}}},\sigma }{{{{{{{\boldsymbol{\delta }}}}}}}}\left(i{c}_{l+\delta,\sigma }^{{{{\dagger}}} }{c}_{l,\sigma }+{{{{{{{\rm{h.c.}}}}}}}}\right)\\ 	+\frac{{t}^{{\prime} }}{2}\mathop{\sum}\limits_{l,{{{{{{{{\boldsymbol{\delta }}}}}}}}}^{{\prime} }\in {{{{{{{\rm{NNN}}}}}}}},\sigma }{{{{{{{{\boldsymbol{\delta }}}}}}}}}^{{\prime} }\left(i{c}_{l+{\delta }^{{\prime} },\sigma }^{{{{\dagger}}} }{c}_{l,\sigma }+{{{{{{{\rm{h.c.}}}}}}}}\right)$$

and

4 $${{{{{{{{\bf{J}}}}}}}}}_{E}=	 \mathop{\sum}\limits_{l,{{{{{{{{\boldsymbol{\delta }}}}}}}}}_{1}\in {{{{{{{\rm{NN}}}}}}}},\atop {{{{{{{{\boldsymbol{\delta }}}}}}}}}_{2}\in {{{{{{{\rm{NN}}}}}}}},\sigma }\left(-\frac{{{{{{{{{\boldsymbol{\delta }}}}}}}}}_{1}+{{{{{{{{\boldsymbol{\delta }}}}}}}}}_{2}}{4}\right){t}^{2}\left(i{c}_{l+{\delta }_{1}+{\delta }_{2},\sigma }^{{{{\dagger}}} }{c}_{l,\sigma }+{{{{{{{\rm{h.c.}}}}}}}}\right)\\ 	+\mathop{\sum}\limits_{l,{{{{{{{\boldsymbol{\delta }}}}}}}}\in {{{{{{{\rm{NN}}}}}}}},\atop {{{{{{{{\boldsymbol{\delta }}}}}}}}}^{{\prime} }\in {{{{{{{\rm{NNN}}}}}}}},\sigma }\left(-\frac{{{{{{{{\boldsymbol{\delta }}}}}}}}+{{{{{{{{\boldsymbol{\delta }}}}}}}}}^{{\prime} }}{2}\right)t{t}^{{\prime} }\left(i{c}_{l+\delta+{\delta }^{{\prime} },\sigma }^{{{{\dagger}}} }{c}_{l,\sigma }+{{{{{{{\rm{h.c.}}}}}}}}\right)\\ 	+\mathop{\sum}\limits_{l,{{{{{{{{\boldsymbol{\delta }}}}}}}}}_{1}^{{\prime} }\in {{{{{{{\rm{NNN}}}}}}}},\atop {{{{{{{{\boldsymbol{\delta }}}}}}}}}_{2}^{{\prime} }\in {{{{{{{\rm{NNN}}}}}}}},\sigma }\left(-\frac{{{{{{{{{\boldsymbol{\delta }}}}}}}}}_{1}^{{\prime} }+{{{{{{{{\boldsymbol{\delta }}}}}}}}}_{2}^{{\prime} }}{4}\right){t}^{{\prime} 2}\left(i{c}_{l+{\delta }_{1}^{{\prime} }+{\delta }_{2}^{{\prime} },\sigma }^{{{{\dagger}}} }{c}_{l,\sigma }+{{{{{{{\rm{h.c.}}}}}}}}\right)\\ 	+\frac{Ut}{4}\mathop{\sum}\limits_{l,{{{{{{{\boldsymbol{\delta }}}}}}}}\in {{{{{{{\rm{NN}}}}}}}},\sigma }{{{{{{{\boldsymbol{\delta }}}}}}}}\left({n}_{l+\delta,-\sigma }+{n}_{l,-\sigma }\right)\left(i{c}_{l+\delta,\sigma }^{{{{\dagger}}} }{c}_{l,\sigma }+{{{{{{{\rm{h.c.}}}}}}}}\right)\\ 	+\frac{U{t}^{{\prime} }}{4}\mathop{\sum}\limits_{l,\sigma,\atop {{{{{{{{\boldsymbol{\delta }}}}}}}}}^{{\prime} }\in {{{{{{{\rm{NNN}}}}}}}}}{{{{{{{{\boldsymbol{\delta }}}}}}}}}^{{\prime} }\left({n}_{l+{\delta }^{{\prime} },-\sigma }+{n}_{l,-\sigma }\right)\left(i{c}_{l+{\delta }^{{\prime} },\sigma }^{{{{\dagger}}} }{c}_{l,\sigma }+{{{{{{{\rm{h.c.}}}}}}}}\right)\\ 	 -\frac{Ut}{4}\mathop{\sum}\limits_{l,{{{{{{{\boldsymbol{\delta }}}}}}}}\in {{{{{{{\rm{NN}}}}}}}},\sigma }{{{{{{{\boldsymbol{\delta }}}}}}}}\left(i{c}_{l+\delta,\sigma }^{{{{\dagger}}} }{c}_{l,\sigma }+{{{{{{{\rm{h.c.}}}}}}}}\right)\\ 	 -\frac{U{t}^{{\prime} }}{4}\mathop{\sum}\limits_{l,{{{{{{{{\boldsymbol{\delta }}}}}}}}}^{{\prime} }\in {{{{{{{\rm{NNN}}}}}}}},\sigma }{{{{{{{{\boldsymbol{\delta }}}}}}}}}^{{\prime} }\left(i{c}_{l+{\delta }^{{\prime} },\sigma }^{{{{\dagger}}} }{c}_{l,\sigma }+{{{{{{{\rm{h.c.}}}}}}}}\right).$$

To make the notations above clear, NN (NNN) denotes the set of nearest-neighbor (next-nearest-neighbor) position displacements. Specifically, on the two-dimensional square lattice, NN = {+**x**, −**x**, +**y**, −**y**} and NNN = {+**x** + **y**, − **x** + **y**, + **x** − **y**, −**x** − **y**}, where the lattice constant is set to 1 and **x** and **y** are unit vectors. Here, if *l* is an arbitrary site label associated with the position vector *x*_*l*_**x** + *y*_*l*_**y**, and ***ν*** is a vector adding up arbitrary elements in NN and NNN, the notation *l* + *ν* represents a unique site label associated with the position *x*_*l*_**x** + *y*_*l*_**y** + ***ν***. The heat current is **J**_*Q*_ = **J**_*E*_ − *μ***J**.

We calculate the thermopower

5 $$S=-\frac{{L}_{{J}_{Q,x}{J}_{x}}}{eT{L}_{{J}_{x}{J}_{x}}}$$

using DQMC and MaxEnt^31,32^. Here, *J*_*Q*,*x*_ and *J*_*x*_ are the *x*-components of the heat current operator **J**_*Q*_ and particle current operator **J**, respectively. For arbitrary Hermitian operators *O*_1_ and ${O}_{2},$ the DC transport coefficient ${L}_{{O}_{1}{O}_{2}} \equiv {\left.{L}_{{O}_{1}{O}_{2}}(\omega )\right|}_{\omega=0}$, where ${L}_{{O}_{1}{O}_{2}}(\omega )$ is determined using the Kubo formula

6 $${L}_{{O}_{1}{O}_{2}}(\omega )=\frac{1}{{N}_{x}{N}_{y}\beta }\int\nolimits_{0}^{\infty }dt{e}^{i(\omega+i{0}^{+})t}\int\nolimits_{0}^{\beta }d\tau \langle {O}_{1}(t-i\tau ){O}_{2}(0)\rangle,$$

where *t* is real time, without confusion with the hopping matrix elements in the Hamiltonian. Here, *N*_*x*_, *N*_*y*_ are the sizes of the lattice along the *x* and *y* directions, respectively, $\beta \equiv {({k}_{B}T)}^{-1}$, and

7 $${O}_{1}(t-i\tau )={e}^{i(H-\mu N)(t-i\tau )}{O}_{1}{e}^{-i(H-\mu N)(t-i\tau )}.$$

Detailed derivations for Eqs. (5) and (2) are in Supplementary Note 2 and Supplementary Note 4, respectively. For our calculation, the units for both *S* and *S*_Kelvin_ are *k*_*B*_/*e* ≈ 86.17 *μV*/*K*.

### Supplementary information

Supplementary Information

Peer Review File

## Data Availability

The data needed to reproduce the figures can be found at 10.5281/zenodo.8286640.

## Code availability

The source code and analysis routines can be found at 10.5281/zenodo.8286636.

## References

[1] Chen Z (2021). Anomalously strong near-neighbor attraction in doped 1D cuprate chains. Science.

[2] Wang Y (2021). Phonon-mediated long-range attractive interaction in one-dimensional cuprates. Phys. Rev. Lett..

[3] Tang T, Moritz B, Peng C, Shen Z-X, Devereaux TP (2023). Traces of electron-phonon coupling in one-dimensional cuprates. Nat. Commun..

[4] Dagotto E (1994). Correlated electrons in high-temperature superconductors. Rev. Mod. Phys..

[5] Arovas DP, Berg E, Kivelson SA, Raghu S (2022). The Hubbard model. Annu. Rev. Condens. Matter Phys..

[6] Qin M, Schäfer T, Andergassen S, Corboz P, Gull E (2022). The Hubbard model: a computational perspective. Annu. Rev. Condens. Matter Phys..

[7] Blankenbecler R, Scalapino DJ, Sugar RL (1981). Monte Carlo calculations of coupled boson-fermion systems. i. Phys. Rev. D..

[8] White SR (1989). Numerical study of the two-dimensional Hubbard model. Phys. Rev. B.

[9] Cooper JR, Alavi B, Zhou L-W, Beyermann WP, Grüner G (1987). Thermoelectric power of some high-*T*_*c*_ oxides. Phys. Rev. B.

[10] Rao CNR, Ramakrishnan TV, Kumar N (1990). Systematics in the thermopower behaviour of several series of bismuth and thallium cuprate superconductors: An interpretation of the temperature variation and the sign of the thermopower. Phys. C: Supercond..

[11] Obertelli SD, Cooper JR, Tallon JL (1992). Systematics in the thermoelectric power of high-*T*_*c*_ oxides. Phys. Rev. B.

[12] Tallon JL, Bernhard C, Shaked H, Hitterman RL, Jorgensen JD (1995). Generic superconducting phase behavior in high-*T*_*c*_ cuprates: *T*_*c*_ variation with hole concentration in YBa_2_ Cu_3_ O_7-δ_. Phys. Rev. B.

[13] Kaiser AB, Subramaniam CK, Ruck B, Paranthaman M (1995). Systematic thermopower behaviour in superconductors. Synth. Met..

[14] Choi M-Y, Kim JS (1999). Thermopower of high-*T*_*c*_ cuprates. Phys. Rev. B.

[15] Honma T, Hor PH (2008). Unified electronic phase diagram for hole-doped high-*T*_*c*_ cuprates. Phys. Rev. B.

[16] Benseman TM, Cooper JR, Zentile CL, Lemberger L, Balakrishnan G (2011). Valency and spin states of substituent cations in Bi_2.15_Sr_1.85_CaCu_2_O_8+*δ*_. Phys. Rev. B.

[17] Zlatić V, Boyd GR, Freericks JK (2014). Universal thermopower of bad metals. Phys. Rev. B.

[18] Newns DM (1994). Quasiclassical transport at a van hove singularity in cuprate superconductors. Phys. Rev. Lett..

[19] McIntosh GC, Kaiser AB (1996). van hove scenario and thermopower behavior of the high-*T*_*c*_ cuprates. Phys. Rev. B.

[20] Chen K-S (2011). Role of the van hove singularity in the quantum criticality of the Hubbard model. Phys. Rev. B.

[21] Mukerjee S, Moore JE (2007). Doping dependence of thermopower and thermoelectricity in strongly correlated materials. Appl. Phys. Lett..

[22] Beni G (1974). Thermoelectric power of the narrow-band Hubbard chain at arbitrary electron density: Atomic limit. Phys. Rev. B.

[23] Chaikin PM, Beni G (1976). Thermopower in the correlated hopping regime. Phys. Rev. B.

[24] Mukerjee S (2005). Thermopower of the Hubbard model: Effects of multiple orbitals and magnetic fields in the atomic limit. Phys. Rev. B.

[25] Phillips P, Choy T-P, Leigh RG (2009). Mottness in high-temperature copper-oxide superconductors. Rep. Prog. Phys..

[26] Chakraborty S, Galanakis D, Phillips P (2010). Emergence of particle-hole symmetry near optimal doping in high-temperature copper oxide superconductors. Phys. Rev. B.

[27] Mousatov CH, Esterlis I, Hartnoll SA (2019). Bad metallic transport in a modified Hubbard model. Phys. Rev. Lett..

[28] Peterson MR, Shastry BS (2010). Kelvin formula for thermopower. Phys. Rev. B.

[29] Garg A, Shastry BS, Dave KB, Phillips P (2011). Thermopower and quantum criticality in a strongly interacting system: parallels with the cuprates. N. J. Phys..

[30] Arsenault L-F, Shastry BS, Sémon P, Tremblay A-MS (2013). Entropy, frustration, and large thermopower of doped Mott insulators on the fcc lattice. Phys. Rev. B.

[31] Jarrell M, Gubernatis JE (1996). Bayesian inference and the analytic continuation of imaginary-time quantum Monte Carlo data. Phys. Rep..

[32] Gunnarsson O, Haverkort MW, Sangiovanni G (2010). Analytical continuation of imaginary axis data for optical conductivity. Phys. Rev. B.

[33] Shastry BS (2008). Electrothermal transport coefficients at finite frequencies. Rep. Prog. Phys..

[34] Paiva T, Scalettar RT, Huscroft C, McMahan AK (2001). Signatures of spin and charge energy scales in the local moment and specific heat of the half-filled two-dimensional Hubbard model. Phys. Rev. B.

[35] Duffy D, Moreo A (1997). Specific heat of the two-dimensional Hubbard model. Phys. Rev. B.

[36] Khatami E, Rigol M (2012). Effect of particle statistics in strongly correlated two-dimensional Hubbard models. Phys. Rev. A.

[37] Wang, W. O. et al. The Wiedemann-Franz law in doped Mott insulators without quasiparticles. *arXiv:* https://arxiv.org/pdf/2208.09144.pdf (2022). 10.1126/science.ade3232 38033050

[38] Wang WO, Ding JK, Moritz B, Huang EW, Devereaux TP (2022). Magnon heat transport in a two-dimensional Mott insulator. Phys. Rev. B.

