# Supplementary material for: Quantitative assessment of the universal thermopower in the Hubbard model
Source: Nat Commun. 2023 Nov 3;14:7064. doi: 10.1038/s41467-023-42772-8 (PMC10624669; doi:10.1038/s41467-023-42772-8)
Supplement: Supplementary file 1 — Supplementary Information [file 41467_2023_42772_MOESM1_ESM.pdf]

Supplementary Information for “Quantitative assessment of the universal thermopower in the Hubbard model”

Wen O. Wang,[1,2,*] Jixun K. Ding,[1,2] Edwin W. Huang,[3,4,5] Brian Moritz,[2] and Thomas P. Devereaux[2,6,7,†]

[1]Department of Applied Physics, Stanford University, Stanford, CA 94305, USA
[2]Stanford Institute for Materials and Energy Sciences, SLAC National Accelerator Laboratory, 2575 Sand Hill Road, Menlo Park, CA 94025, USA
[3]Department of Physics and Institute of Condensed Matter Theory, University of Illinois at Urbana-Champaign, Urbana, IL 61801, USA
[4]Department of Physics and Astronomy, University of Notre Dame, Notre Dame, IN 46556, USA
[5]Stavropoulos Center for Complex Quantum Matter, University of Notre Dame, Notre Dame, IN 46556, USA
[6]Department of Materials Science and Engineering, Stanford University, Stanford, CA 94305, USA
[7]Geballe Laboratory for Advanced Materials, Stanford University, Stanford, CA 94305, USA

(Dated: October 31, 2023)

Supplementary Note 1: Simulation parameters

Statistical error bars denoting ± 1 standard error of the mean are shown for all measurements, except for Supplementary Fig. 2 that has none. Error bars are determined by bootstrap resampling (100 bootstraps) [1], except for error bars determined by jackknife resampling [2]: n in the inset of Fig. 2b in the main text, S_{Kelvin} in Supplementary Fig. 3, and 16×16 S_{Kelvin} data in Supplementary Fig. 7b. Simulation cluster size is 8×8 for all results, unless otherwise specified. The maximum imaginary time Trotter discretization is $d\tau = 0.02/t$ in the chemical potential tuning process, and $d\tau = 0.05/t$ for other thermodynamic and transport measurements, unless otherwise specified. At high temperatures, the smallest number of imaginary-time slices used in the Trotter decomposition is $\tilde{L} = \beta/d\tau = 20$. For MaxEnt analytic continuation, we choose the model function by using the same high-temperature annealing procedure as in Ref. [3], except for Supplementary Fig. 1. We determine spectra in the infinite-temperature-limit, using a moments expansion method, which serves as the model function at the highest temperature, except for Supplementary Fig. 2, similar as in Refs. [3–5]. To determine the adjustable parameter which assigns weights of statistics and entropy in the maximized function in MaxEnt, we use the method of Ref. [6]. Other details in methods and parameter choices are mostly the same as Ref. [3].

Supplementary Note 2: Formalism

We set $\hbar$ to 1 throughout the paper. We consider the response due to a temperature gradient ∇T and electric field $\mathbf{E} = -\nabla V$. We define $\overline{\mu} = \mu + e^* V$ so that $\nabla\overline{\mu} = \nabla\mu - e^*\mathbf{E}$, where charge $e^* = -e$ for electrons. The responses along the x direction in terms of DC transport coefficients $L_{O_1 O_2}$ ($\omega = 0$ value of Eq. (6) in the main text) are [5, 7]

$$\begin{aligned}\langle J_x\rangle/(N_x N_y) &= -\beta L_{J_x J_x}\partial_x\overline{\mu} + L_{J_x J_{Q,x}}\partial_x\beta \\ &= -\beta L_{J_x J_x}\partial_x\overline{\mu} - L_{J_x J_{Q,x}}\beta^2 k_B \partial_x T, \quad (1)\\ \langle J_{Q,x}\rangle/(N_x N_y) &= -\beta L_{J_{Q,x} J_x}\partial_x\overline{\mu} + L_{J_{Q,x} J_{Q,x}}\partial_x\beta \\ &= -\beta L_{J_{Q,x} J_x}\partial_x\overline{\mu} - L_{J_{Q,x} J_{Q,x}}\beta^2 k_B \partial_x T. \quad (2)\end{aligned}$$

The thermopower S is defined as

$$S = -\left.\frac{\partial_x\overline{\mu}}{e^*\partial_x T}\right|_{\langle J_x\rangle=0} = -\frac{L_{J_x J_{Q,x}}}{eTL_{J_x J_x}} = -\frac{L_{J_{Q,x} J_x}}{eTL_{J_x J_x}}, \quad (3)$$

giving us Eq. (5) in the main text. In Supplementary Eq. (3), we used Onsager's reciprocity relations [8]

$$L_{J_x J_{Q,x}} = L_{J_{Q,x} J_x}. \quad (4)$$

Setting $Z = \mathrm{Tr}(e^{-\beta(H-\mu N)})$ as the partition function, from Eq. (6) in the main text,

$$\begin{aligned}L_{O_1 O_2}(\omega) = &\frac{1}{ZN_x N_y \beta}\sum_{i_1,i_2}\langle i_1|O_1|i_2\rangle\langle i_2|O_2|i_1\rangle \\ &\times \frac{e^{-\beta E_{i_1}} - e^{-\beta E_{i_2}}}{i(E_{i_1}-E_{i_2})(\omega + i0^+ + E_{i_1} - E_{i_2})}. \quad (5)\end{aligned}$$

In the case of $O_1 = O_2 = O$, we obtain

$$\begin{aligned}\mathrm{Re}\, L_{OO}(\omega) = &\frac{\pi}{ZN_x N_y \beta\omega}\sum_{i_1,i_2}|\langle i_1|O|i_2\rangle|^2 \\ &\times e^{-\beta E_{i_1}}(1 - e^{-\beta\omega})\delta(\omega + E_{i_1} - E_{i_2}), \quad (6)\end{aligned}$$

where $|i_i\rangle$ (E_{i_1}) are eigenstates (eigenvalues) of the grand-canonical Hamiltonian $H - \mu N$. From Supplementary Eq. (6) we obtain $\mathrm{Re}\, L_{OO}(\omega) = \mathrm{Re}\, L_{OO}(-\omega)$. By Kramers-Kronig relations, $\mathrm{Im}\, L_{OO}(\omega = 0) = 0$.

We use DQMC to measure correlation functions in imaginary time,

$$\begin{aligned}&\langle T_\tau O_1(\tau) O_2(0)\rangle \\ &\equiv \frac{1}{Z}\mathrm{Tr}\left(e^{-(\beta-\tau)(H-\mu N)} O_1 e^{-\tau(H-\mu N)} O_2\right) \\ &= \frac{1}{Z}\sum_{i_1,i_2}\langle i_1|O_1|i_2\rangle\langle i_2|O_2|i_1\rangle e^{-\beta E_{i_1}} e^{\tau(E_{i_1}-E_{i_2})}. \quad (7)\end{aligned}$$

* wenwang.physics@gmail.com
† tpd@stanford.edu

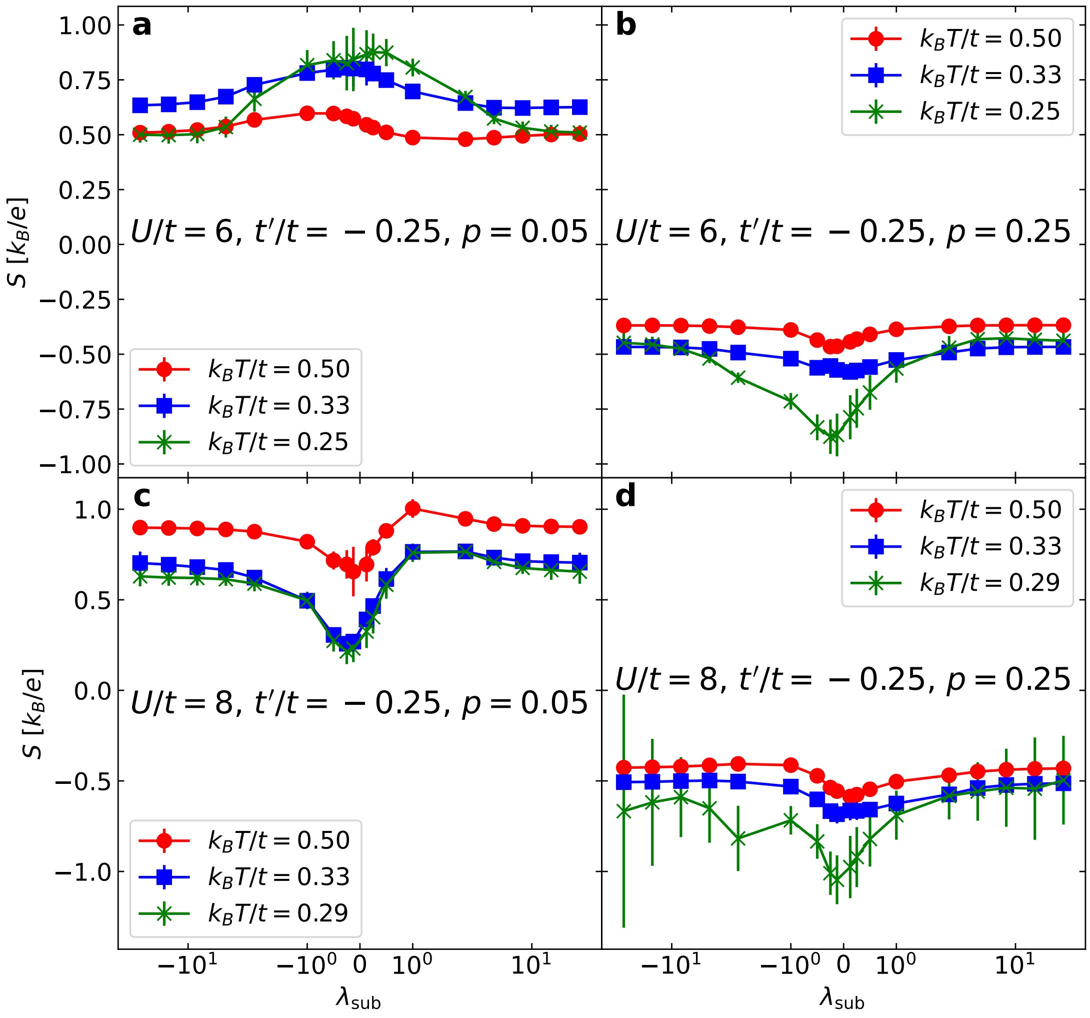

Supplementary Fig. 1. S as a function of $\lambda_{\rm sub}$ at a few different temperatures, for four sets of representative parameters, all with $t'/t = -0.25$: (**a**) $U/t = 6$, $p = 0.05$, (**b**) $U/t = 6$, $p = 0.25$, (**c**) $U/t = 8$, $p = 0.05$, and (**d**) $U/t = 8$, $p = 0.25$. For MaxEnt analytic continuation, a flat model function is used for all parameters.

Comparing Supplementary Eqs. (6) and (7), we relate $\mathrm{Re}\, L_{OO}(\omega)$ with $\langle T_\tau O(\tau) O(0)\rangle$ through

$$\frac{\langle T_\tau O(\tau)O(0)\rangle}{N_x N_y \beta} = \int_0^\infty d\omega\, \mathrm{Re}\, L_{OO}(\omega) \frac{\omega \cosh[\omega(\tau - \beta/2)]}{\pi \sinh[\beta\omega/2]}. \quad (8)$$

We apply MaxEnt analytic continuation to $\langle T_\tau O(\tau)O(0)\rangle$ data to invert Supplementary Eq. (8) and obtain $\mathrm{Re}\, L_{OO}(\omega)$.

According to Eq. (6) in the main text, we may write

$$\frac{1}{2}(L_{O_1O_2}(\omega) + L_{O_2O_1}(\omega)) = \big(L_{(\lambda_{\rm sub}O_1+O_2)(\lambda_{\rm sub}O_1+O_2)}(\omega) - \lambda_{\rm sub}^2 L_{O_1O_1}(\omega) - L_{O_2O_2}(\omega)\big)/(2\lambda_{\rm sub}), \quad (9)$$

where $\lambda_{\rm sub}$ is an arbitrary non-zero real constant [9]. With Supplementary Eq. (9), Supplementary Eq. (8) can be generalized,

$$\frac{\langle T_\tau O_1(\tau)O_2(0)\rangle + \langle T_\tau O_2(\tau)O_1(0)\rangle}{N_x N_y \beta} = \int_0^\infty d\omega\, \mathrm{Re}\left[L_{O_1O_2}(\omega) + L_{O_2O_1}(\omega)\right] \frac{\omega \cosh[\omega(\tau-\beta/2)]}{\pi \sinh[\beta\omega/2]}. \quad (10)$$

$\mathrm{Re}\, L_{OO}(\omega)$ is guaranteed to be positive definite in Supplementary Eq. (6) when $O_1 = O_2 = O$, in which case MaxEnt analytic continuation is applicable. However, in calculation of the thermopower, $\mathrm{Re}\, L_{J_{Q,x}J_x}(\omega) + \mathrm{Re}\, L_{J_xJ_{Q,x}}(\omega)$ can change its sign as a function of ω, so it cannot be directly calculated from $\langle T_\tau J_{Q,x}(\tau)J_x\rangle + \langle T_\tau J_x(\tau)J_{Q,x}\rangle$ using Supplementary Eq. (10) through MaxEnt. So, according to Supplementary Eqs. (4) and (9), we calculate $L_{J_{Q,x}J_x}$ using

$$L_{J_{Q,x}J_x} = \big(L_{(\lambda_{\rm sub}J_{Q,x}+J_x)(\lambda_{\rm sub}J_{Q,x}+J_x)} - \lambda_{\rm sub}^2 L_{J_{Q,x}J_{Q,x}} - L_{J_xJ_x}\big)/(2\lambda_{\rm sub}). \quad (11)$$

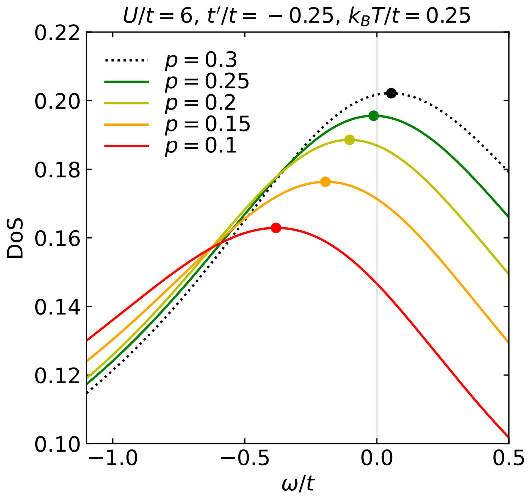

Supplementary Fig. 2. Density of states (DoS) as a function of ω for different dopings, for $U/t = 6$ and $t'/t = -0.25$ at $k_BT/t = 0.25$. $\omega = 0$ (light grey vertical line) sets the Fermi level.

Since $L_{OO} \equiv L_{OO}(\omega)|_{\omega=0}$ is real, $L_{J_{Q,x}J_x}$ is also real. In principle, if there are no errors in every L_{OO} term on the right hand side of Supplementary Eq. (11), then the result of $L_{J_{Q,x}J_x}$ from Supplementary Eq. (11) is $\lambda_{\rm sub}$ independent. However, systematic errors introduced by the analytic continuation process propagate in the calculation of Supplementary Eq. (11), which is reflected by S exhibiting some degree of $\lambda_{\rm sub}$ dependence. In Supplementary Fig. 1, we show S as a function of $\lambda_{\rm sub}$ for four sets of parameters as examples. As long as $|\lambda_{\rm sub}| \gtrsim 1$, the $\lambda_{\rm sub}$ dependence is relatively weak. Therefore, as a reasonable choice, we use $\lambda_{\rm sub} = 2$ in this work.

Supplementary Note 3: Lifshitz transition

We calculate the density of states (DoS) from the DQMC results of the local Green's function $G(\tau) = -\langle T_\tau c_{l,\sigma}(\tau) c^\dagger_{l,\sigma}(0)\rangle$, by inverting the relation [10]

$$G(\tau) = -\int_{-\infty}^{+\infty} d\omega \frac{e^{-\tau\omega}}{1+e^{-\beta\omega}} \mathrm{DoS}(\omega), \tag{12}$$

using MaxEnt analytic continuation. For the model function in MaxEnt, we start with using the flat model at the highest temperature $k_BT/t = 8$, and proceed with lower temperatures using the high-temperature annealing procedure. In Supplementary Fig. 2 we show doping dependence of $\mathrm{DoS}(\omega)$ for fixed $U/t = 6$, $t'/t = -0.25$, and $k_BT/t = 0.25$. We observe that the Lifshitz transition, at which the quasiparticle peak crosses the Fermi level at $\omega = 0$, happens at doping $p \sim 0.26$, which is much higher than the sign change doping of S at $p \sim 0.15$ in Fig. 1 in the main text for the corresponding parameter set. Therefore, the sign change doping of S is not associated with the Lifshitz transition.

Supplementary Note 4: Kelvin formula

The Kelvin formula for thermopower is [11]

$$S_{\rm Kelvin} = \frac{1}{e^*}\left(\frac{\partial s}{\partial n}\right)_T = -\frac{1}{e^*}\left(\frac{\partial \mu}{\partial T}\right)_n, \tag{13}$$

where s is the entropy density and n is the particle density.

To obtain the second equality in Supplementary Eq. (13), we consider the thermodynamic potential density $f = \epsilon - sT - \mu n$, where ϵ is the energy density. Using the first law of thermodynamics,

$$d\epsilon = T\,ds + \mu\,dn, \tag{14}$$

we obtain $d(f + \mu n) = -s\,dT + \mu\,dn$. Equating

$$\frac{\partial^2(f+\mu n)}{\partial T \partial n} = \frac{\partial^2(f+\mu n)}{\partial n \partial T} \tag{15}$$

then gives us the Maxwell relation leading to the second equality in Supplementary Eq. (13).

From Supplementary Eqs. (13) and (14), we find

$$S_{\rm Kelvin} = \frac{1}{e^*}\left[\frac{1}{T}\left(\frac{\partial \epsilon}{\partial n}\right)_T - \frac{\mu}{T}\right], \tag{16}$$

where

$$\left(\frac{\partial \epsilon}{\partial n}\right)_T = \left(\frac{\partial \epsilon}{\partial \mu}\right)_T \Big/ \left(\frac{\partial n}{\partial \mu}\right)_T. \tag{17}$$

In terms of correlation functions, which we measure using DQMC,

$$\left(\frac{\partial \epsilon}{\partial \mu}\right)_T = \frac{\partial}{\partial \mu}\frac{\mathrm{Tr}\, He^{-\beta(H-\mu N)}}{N_xN_y\,\mathrm{Tr}\, e^{-\beta(H-\mu N)}} = \frac{\beta}{N_xN_y}(\langle HN\rangle - \langle H\rangle\langle N\rangle), \tag{18}$$

$$\left(\frac{\partial n}{\partial \mu}\right)_T = \frac{\partial}{\partial \mu}\frac{\mathrm{Tr}\, Ne^{-\beta(H-\mu N)}}{N_xN_y\,\mathrm{Tr}\, e^{-\beta(H-\mu N)}} = \frac{\beta}{N_xN_y}(\langle NN\rangle - \langle N\rangle\langle N\rangle). \tag{19}$$

Taking Supplementary Eqs. (16), (17), (18), and (19), with $e^* = -e$, we obtain Eq. (2) in the main text.

The specific heat (considering Supplementary Eq. (14)) is

$$c_v = \left(\frac{\partial \epsilon}{\partial T}\right)_n = T\left(\frac{\partial s}{\partial T}\right)_n. \tag{20}$$

So from Supplementary Eqs. (13) and (20), we obtain

$$e^*\left(\frac{\partial S_{\rm Kelvin}}{\partial T}\right)_n = \frac{\partial^2 s}{\partial n \partial T} = \frac{\partial^2 s}{\partial T \partial n} = \frac{1}{T}\left(\frac{\partial c_v}{\partial n}\right)_T. \tag{21}$$

Therefore, the temperature dependence of $S_{\rm Kelvin}$ is directly related to doping dependence of the specific heat c_v. In the

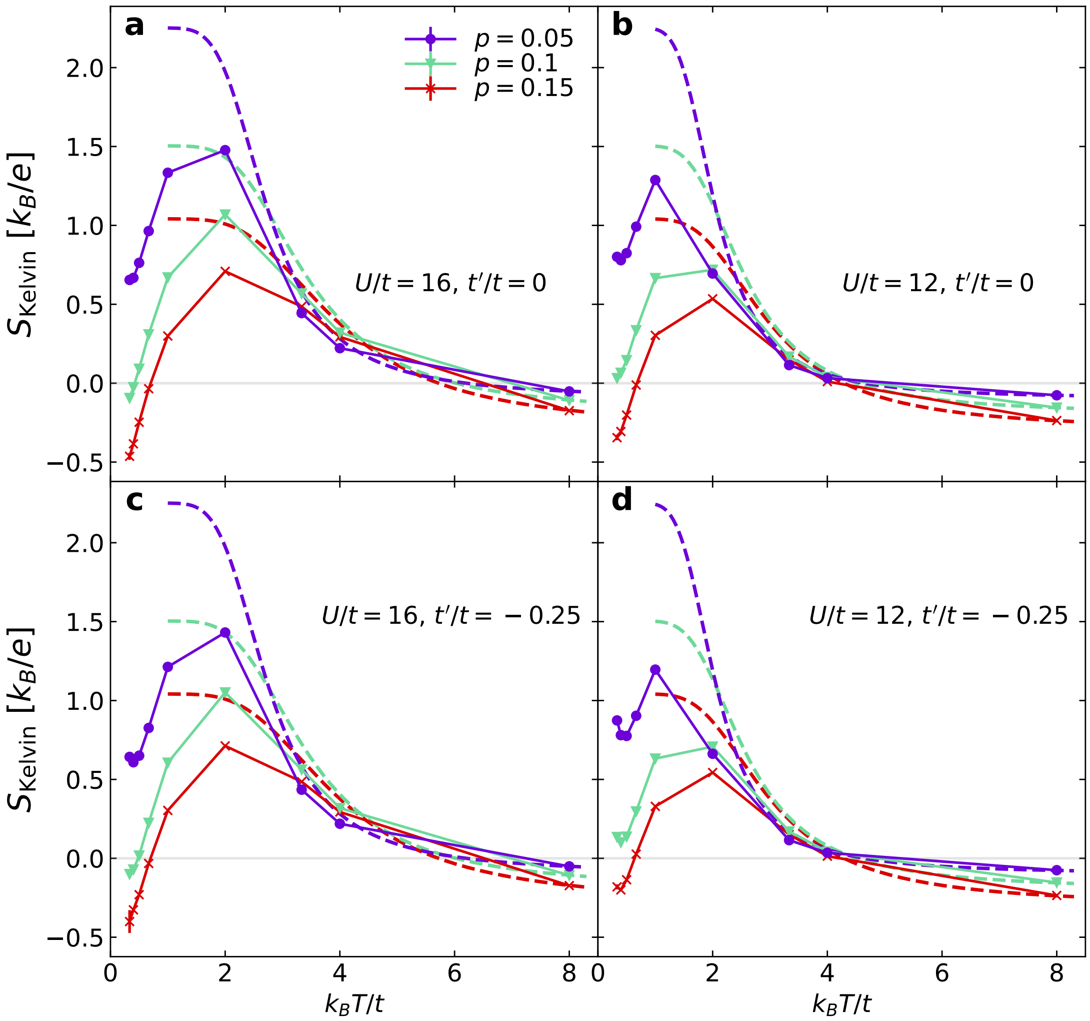

Supplementary Fig. 3. Comparison between Hubbard model (solid lines, obtained by DQMC) and atomic-limit (dashed lines, from Supplementary Eq. (25)) results of S_{Kelvin} for large interactions: (**a**) $U/t = 16, t'/t = 0$, (**b**) $U/t = 12, t'/t = 0$, (**c**) $U/t = 16, t'/t = -0.25$, and (**d**) $U/t = 12, t'/t = -0.25$. For these parameters, the maximum $d\tau$ for chemical potential tuning is $0.01/t$. Error bars denote ± 1 standard error of the mean determined by jackknife resampling.

main text, we use doping $p = 1 - n$ instead of n. So we rewrite Supplementary Eq. (21) as

$$-e\left(\frac{\partial S_{\mathrm{Kelvin}}}{\partial T}\right)_p = -\frac{\partial^2 s}{\partial p \partial T} = -\frac{\partial^2 s}{\partial T \partial p} = -\frac{1}{T}\left(\frac{\partial c_v}{\partial p}\right)_T . \tag{22}$$

For the calculation of $-\partial^2 s/(\partial p \partial T)$ in Fig. 4 in the main text, to rule out data points with large error bars in the spline fitting process, for the fitting of c_v, the lowest temperature considered is $k_BT = t/3.5$ for $U/t = 6$ and $k_BT = t/3$ for $U/t = 8$; for S_{Kelvin}, the lowest temperature in the fitting range is $k_BT = t/4.5$ for $U/t = 6$ and $k_BT = t/3.5$ for $U/t = 8$. Since the measurements of c_v involve energy fluctuation and therefore contains correlators with up to 8 fermion operators, while S_{Kelvin} contains up to 6, for the same set of parameters, c_v data generally has larger statistical error than S_{Kelvin}. Therefore a higher lowest temperature is chosen for fitting c_v than that for S_{Kelvin}.

Supplementary Note 5: Atomic limit

In this note we derive the atomic-limit ($t, t' \ll k_BT, U$) approximation of S and S_{Kelvin}.

Considering the condition $t, t' \ll U$, we divide the Hamiltonian of Eq. (1) in the main text into the interaction part $H_0 \propto U$ as the unperturbed Hamiltonian and the kinetic part ΔH as the perturbative term. Namely,

$$\begin{aligned} H_0 &= U \sum_l \left(n_{l,\uparrow} - \frac{1}{2}\right)\left(n_{l,\downarrow} - \frac{1}{2}\right), \\ \Delta H &= -t \sum_{\langle lm \rangle, \sigma} \left(c^\dagger_{l,\sigma} c_{m,\sigma} + \mathrm{h.c.}\right) \\ &\quad - t' \sum_{\langle\langle lm \rangle\rangle, \sigma} \left(c^\dagger_{l,\sigma} c_{m,\sigma} + \mathrm{h.c.}\right). \end{aligned}$$

By expanding

$$e^{-\tau(H-\mu N)} = e^{-\tau(H_0-\mu N)}\left[1 - \int_0^\tau d\tau_1 \Delta H(\tau_1) + \int_0^\tau d\tau_1 \int_0^{\tau_1} d\tau_2 \Delta H(\tau_1)\Delta H(\tau_2) + ...\right], \quad (23)$$

where $\Delta H(\tau_1) = e^{\tau_1(H_0-\mu N)}\Delta H e^{-\tau_1(H_0-\mu N)}$, the $O_1 - O_2$ correlation function between arbitrary Hermitian operators O_1, O_2 is

$$\begin{aligned}&\langle T_\tau O_1(\tau) O_2\rangle \\ &= \frac{\mathrm{Tr}\left(e^{-(\beta-\tau)(H-\mu N)} O_1 e^{-\tau(H-\mu N)} O_2\right)}{\mathrm{Tr}\, e^{-\beta(H-\mu N)}} \\ &= \frac{\mathrm{Tr}\big(e^{-(\beta-\tau)(H_0-\mu N)} O_1 e^{-\tau(H_0-\mu N)} O_2\big)}{\mathrm{Tr}\, e^{-\beta(H_0-\mu N)}}(1+\mathcal{O}(\beta t)).\end{aligned} \quad (24)$$

Using Supplementary Eq. (24) evaluated under the occupation basis (the eigenstates of H_0), Eq. (2) in the main text can be obtained to leading order. This leads to the atomic-limit approximation

$$S_{\mathrm{Kelvin}} = \frac{-U\left(e^{2\beta\mu+\frac{\beta U}{2}} + e^{\beta\mu}\right)}{eT\left(e^{\frac{\beta U}{2}} + e^{2\beta\mu+\frac{\beta U}{2}} + 2e^{\beta\mu}\right)} + \frac{\frac{U}{2}+\mu}{eT}. \quad (25)$$

In the same limit, we can calculate the average density $\langle n\rangle$. Applying Supplementary Eq. (23), we find

$$\begin{aligned}\langle n\rangle &= \frac{\mathrm{Tr}\big(e^{-\beta(H-\mu N)} N\big)}{N_x N_y \,\mathrm{Tr}\big(e^{-\beta(H-\mu N)}\big)} \\ &= \frac{\mathrm{Tr}\big(e^{-\beta(H_0-\mu N)} N\big)}{N_x N_y \,\mathrm{Tr}\big(e^{-\beta(H_0-\mu N)}\big)}(1+\mathcal{O}(\beta t)).\end{aligned} \quad (26)$$

Therefore, to leading order,

$$\langle n\rangle = \frac{2e^{\frac{1}{2}\beta U+\beta\mu} + 2e^{2\beta\mu}}{1+2e^{\frac{1}{2}\beta U+\beta\mu} + e^{2\beta\mu}}, \quad (27)$$

which allows us to determine μ for any given density n in the atomic limit.

In Supplementary Fig. 3, we compare S_{Kelvin} calculated using DQMC with the atomic-limit approximation of S_{Kelvin}, Supplementary Eq. (25). Large interactions $U/t = 16$ and $U/t = 12$ are selected. At high temperatures, where the condition $t, t' \ll k_B T$ is satisfied, the simulation results match the atomic-limit approximations well. As temperature decreases and this condition breaks down, S_{Kelvin} deviates from its atomic-limit approximation.

Now, we derive the atomic-limit approximation for thermopower S. Still using the occupation basis, and replacing O_1 and O_2 with J_x or $J_{E,x}$ operators in Supplementary Eq. (24), the $J_{E,x} - J_x$ and $J_x - J_x$ correlation functions to leading order are

$$\frac{\langle T_\tau J_{E,x}(\tau) J_x\rangle}{N_x N_y} = \frac{\mathrm{Tr}\big(e^{-(\beta-\tau)(H_0-\mu N)} J_{E,x} e^{-\tau(H_0-\mu N)} J_x\big)}{N_x N_y \,\mathrm{Tr}\, e^{-\beta(H_0-\mu N)}} \quad (28)$$

$$\begin{aligned}&= \frac{-16t^2t' - 2U(t^2+2t'^2)}{Z_0^2}\times \\ &\left(e^{3\beta\mu+\frac{\beta U}{2}} + e^{\beta\mu+\frac{\beta U}{2}} + e^{2\beta\mu+\tau U} + e^{2\beta\mu+\beta U-\tau U}\right) \\ &+ \frac{2U(t^2+2t'^2)}{Z_0^2}\left(2e^{3\beta\mu+\frac{\beta U}{2}} + e^{2\beta\mu+\tau U} + e^{2\beta\mu+\beta U-\tau U}\right)\end{aligned} \quad (29)$$

$$= \frac{\langle T_\tau J_x(\tau) J_{E,x}\rangle}{N_x N_y}$$

and

$$\begin{aligned}\frac{\langle T_\tau J_x(\tau) J_x\rangle}{N_x N_y} &= \frac{\mathrm{Tr}\big(e^{-(\beta-\tau)(H_0-\mu N)} J_x e^{-\tau(H_0-\mu N)} J_x\big)}{N_x N_y \,\mathrm{Tr}\, e^{-\beta(H_0-\mu N)}} \\ &= \frac{4(t^2+2t'^2)}{Z_0^2}\times \\ &\left(e^{3\beta\mu+\frac{\beta U}{2}} + e^{\beta\mu+\frac{\beta U}{2}} + e^{2\beta\mu+\tau U} + e^{2\beta\mu+\beta U-\tau U}\right),\end{aligned} \quad (30)$$

where $Z_0 = 1 + 2e^{\beta U/2+\beta\mu} + e^{2\beta\mu}$. Notice that any term of the form $(e^{-(\beta-\tau)U} + e^{-\tau U})$ multiplied by a quantity independent of τ in $\langle T_\tau O_1(\tau)O_2\rangle + \langle T_\tau O_2(\tau)O_1\rangle$ corresponds to a delta function at $\omega = U$ in $\mathrm{Re}\, L_{O_1O_2}(\omega) + \mathrm{Re}\, L_{O_2O_1}(\omega)$ through Supplementary Eq. (10). Such terms do not contribute to the DC values of transport coefficients. Any term independent of τ in $\langle T_\tau O_1(\tau)O_2\rangle + \langle T_\tau O_2(\tau)O_1\rangle$ corresponds to a delta function at $\omega = 0$ in $\mathrm{Re}\, L_{O_1O_2}(\omega) + \mathrm{Re}\, L_{O_2O_1}(\omega)$. Summing up magnitudes of such terms provides the integrated weights of $\mathrm{Re}\, L_{O_1O_2}(\omega) + \mathrm{Re}\, L_{O_2O_1}(\omega)$ around $\omega = 0$. So, using finite-frequency Onsager relations [7], Supplementary Eqs. (10), (29), and (30), with $|\tilde{\epsilon}| < U$, we have

$$\begin{aligned}&\frac{1}{\pi}\int_{-|\tilde{\epsilon}|}^{+|\tilde{\epsilon}|} \mathrm{Re}\, L_{J_{E,x}J_x}(\omega)d\omega = \frac{1}{\pi}\int_{-|\tilde{\epsilon}|}^{+|\tilde{\epsilon}|} \mathrm{Re}\, L_{J_xJ_{E,x}}(\omega)d\omega \\ &= \frac{-16t^2t' - 2U(t^2+2t'^2)}{Z_0^2}\left(e^{3\beta\mu+\frac{\beta U}{2}} + e^{\beta\mu+\frac{\beta U}{2}}\right) \\ &+ \frac{4U(t^2+2t'^2)}{Z_0^2} e^{3\beta\mu+\frac{\beta U}{2}},\end{aligned} \quad (31)$$

$$\begin{aligned}&\frac{1}{\pi}\int_{-|\tilde{\epsilon}|}^{+|\tilde{\epsilon}|} \mathrm{Re}\, L_{J_xJ_x}(\omega)d\omega \\ &= \frac{4(t^2+2t'^2)}{Z_0^2}\left(e^{3\beta\mu+\frac{\beta U}{2}} + e^{\beta\mu+\frac{\beta U}{2}}\right).\end{aligned} \quad (32)$$

Here, both $\mathrm{Re}\, L_{J_xJ_x}(\omega)$ and $\mathrm{Re}\, L_{J_{E,x}J_x}(\omega)$ are proportional to $\delta(\omega)$ at low frequencies, so they are both infinite at $\omega = 0$. To make both $\mathrm{Re}\, L_{J_xJ_x}(\omega = 0)$ and $\mathrm{Re}\, L_{J_{E,x}J_x}(\omega = 0)$ finite, we introduce a small scattering rate [12–15], or broadening effect, to both coefficients. The same scattering rate

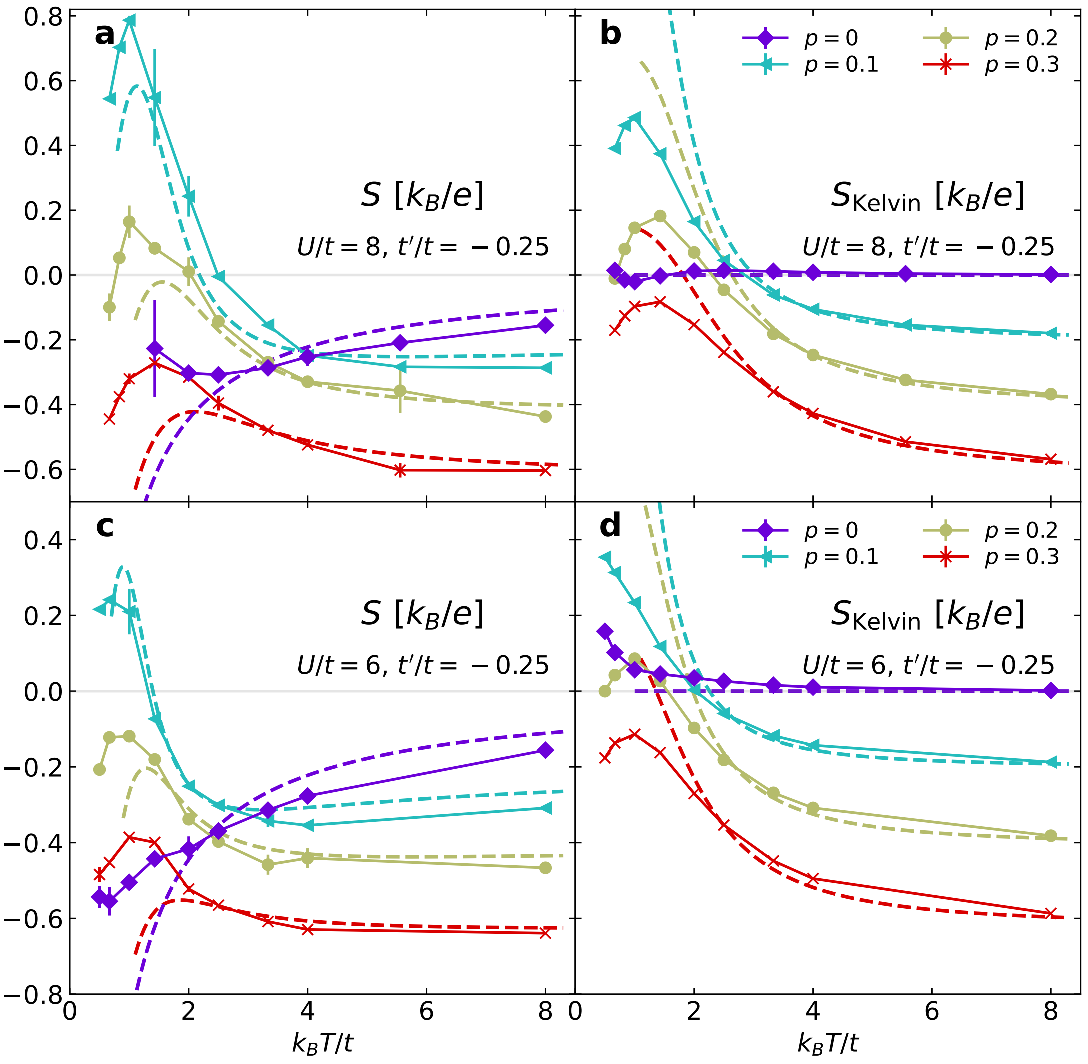

Supplementary Fig. 4. Hubbard model simulation data (solid lines, S obtained with DQMC and MaxEnt, $S_{\rm Kelvin}$ obtained with DQMC) at high temperatures compared with corresponding atomic-limit results (dashed lines, from Supplementary Eqs. (33) and (25)), for $U/t = 8$ in (**a**, **b**), and $U/t = 6$ in (**c**, **d**). All panels have $t'/t = -0.25$.

in both terms cancels out when we take their ratio and gives us the ratio of corresponding weights. Under this assumption, combining Supplementary Eqs. (3), (31), (32), and that $\mathbf{J}_Q = \mathbf{J}_E - \mu\mathbf{J}$, we obtain the atomic-limit approximation of thermopower to leading order,

$$S = \lim_{|\tilde{\epsilon}|\to 0} -\frac{\int_{-|\tilde{\epsilon}|}^{+|\tilde{\epsilon}|} d\omega(\mathrm{Re}\, L_{J_{E,x}J_x}(\omega) - \mu\,\mathrm{Re}\, L_{J_xJ_x}(\omega))}{eT\int_{-|\tilde{\epsilon}|}^{+|\tilde{\epsilon}|} d\omega\,\mathrm{Re}\, L_{J_xJ_x}(\omega)}$$
$$= \frac{4t^2t'(e^{2\beta\mu}+1) - U(t^2+2t'^2)e^{2\beta\mu}}{eT(t^2+2t'^2)(e^{2\beta\mu}+1)} + \frac{\frac{U}{2}+\mu}{eT}. \qquad (33)$$

An interesting observation in the atomic limit is that t and t' affect S (a transport property) in Supplementary Eq. (33), but not $S_{\rm Kelvin}$ (a thermodynamics property) in Supplementary Eq. (25). If we take $t' = 0$, the expression Supplementary Eq. (33) is equivalent to corresponding expressions of S derived and discussed in Refs. [12–15], where the chemical potential is different from our definition by $U/2$ due to the difference in the Hamiltonian definition.

When we additionally impose the conditions $k_BT/U \ll 1$ (i.e. $\beta U \gg 1$) and $n < 1$, and use μ as determined from Supplementary Eq. (27) under these conditions, Supplementary Eq. (25) and the $t' = 0$ case of Supplementary Eq. (33) both approach the "Heikes formula" [12–16]

$$S_{\rm Kelvin} = S = \frac{\frac{U}{2}+\mu}{eT} = \frac{k_B}{e}\ln\left[\frac{n}{2(1-n)}\right]. \qquad (34)$$

This "Heikes limit" in Supplementary Eq. (34) produces a sign change at $p = 1/3$ [12–18].

In Supplementary Fig. 4, we compare Hubbard model simulation results with the atomic limit of S (Supplementary Eq. (33)) and $S_{\rm Kelvin}$ (Supplementary Eq. (25)), for $t'/t = -0.25$ and $U/t = 6$ or 8. The atomic-limit sign-change p of S shifts away from $1/3$ when t' becomes non-zero, because of additional terms introduced by t' in Supplementary

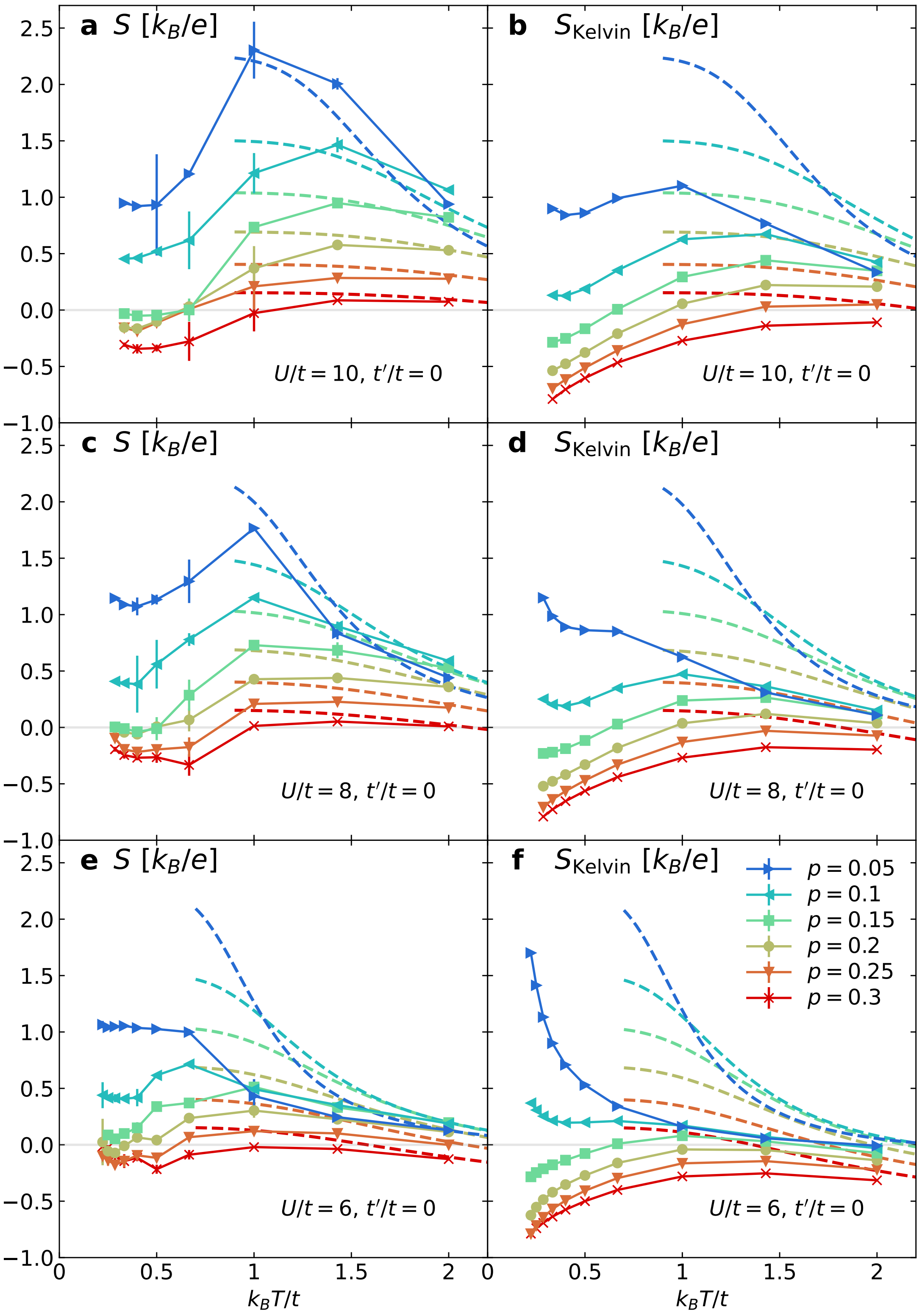

Supplementary Fig. 5. Comparison similar to Supplementary Fig. 4 for $t'/t = 0$, $T \lesssim 2$, (**a**, **b**) $U/t = 10$, (**c**, **d**) $U/t = 8$, and (**e**, **f**) $U/t = 6$.

Eq. (33). Supplementary Figure 5 presents the same comparison as Supplementary Fig. 4, but for $t'/t = 0$ and $U/t = 6$ to 10 and focusing on lower temperatures. In all panels of Supplementary Figs. 4 and 5, we see that simulation results (unsurprisingly) match the atomic-limit approximations at high temperatures but deviate as temperature decreases.

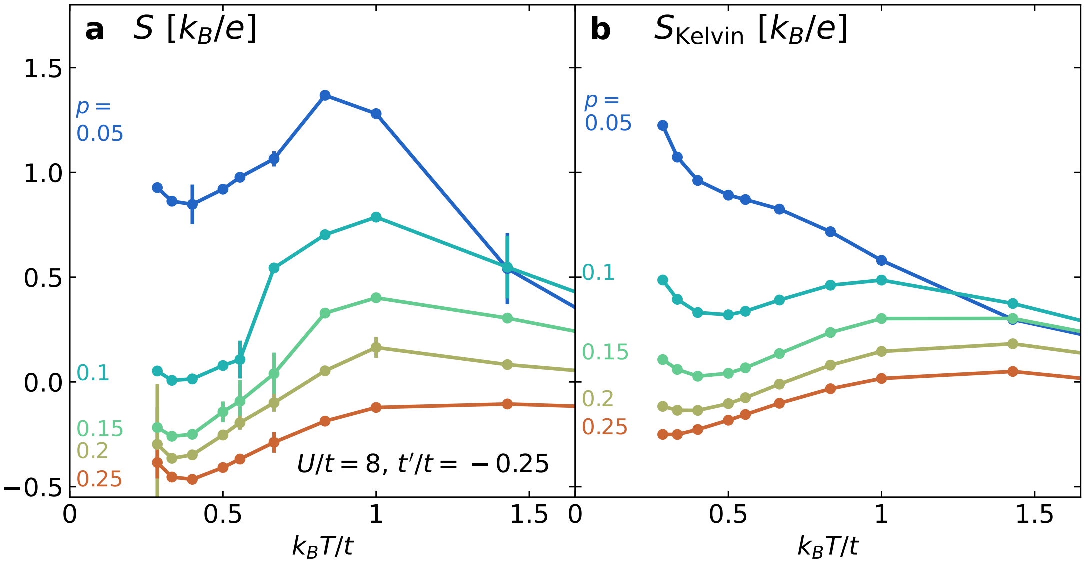

Supplementary Fig. 6. Temperature dependence of S (**a**) and S_{Kelvin} (**b**), plotted in the same way as Fig. 3 in the main text, but for $U/t = 8$ and $t'/t = -0.25$.

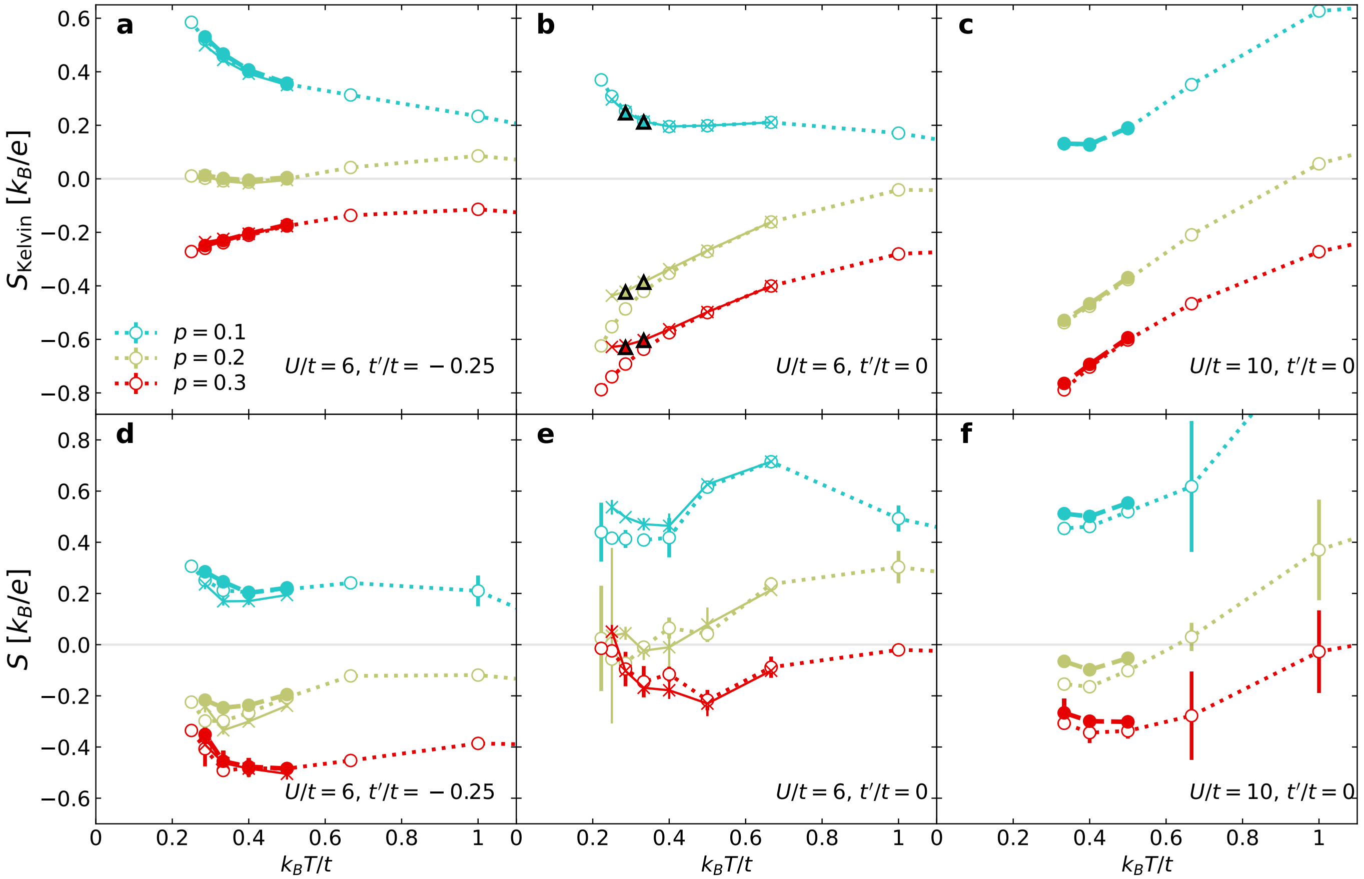

Supplementary Fig. 7. Finite size and Trotter error analysis for S_{Kelvin} (**a**, **b**, **c**) and S (**d**, **e**, **f**). Dotted lines with open circles are obtained with $d\tau = 0.05/t$ on an 8×8 lattice, which are the parameters we use in the main text. Solid lines with crosses are obtained with $d\tau = 0.05/t$ on a 12×12 lattice. Dashed lines with filled circles are obtained with $d\tau = 0.025/t$ on an 8×8 lattice. Triangles in (**b**) are obtained with $d\tau = 0.05/t$ on a 16×16 lattice. For the solid and the dashed lines in (**d**), and the dashed lines in (**f**), the MaxEnt model functions for highest temperature ($\beta = 2/t$) are chosen to be the spectral functions obtained with $d\tau = 0.05/t$ on an 8×8 lattice at $\beta = 1.5/t$. Similarly, for solid lines in (**e**), the MaxEnt model functions for highest temperature ($\beta = 1.5/t$) are chosen to be the spectral functions obtained with $d\tau = 0.05/t$ on an 8×8 lattice at $\beta = 1/t$.

Supplementary Note 6: Supplementary data

For the sake of completeness, we show the temperature dependence of S and S_{Kelvin} for $U/t = 8$ and $t'/t = -0.25$ in Supplementary Fig. 6. We find the behaviors of S and S_{Kelvin} are qualitatively similar to the case of $U/t = 6$ and $t'/t = -0.25$, shown in Fig. 3 in the main text.

Supplementary Note 7: Finite size and Trotter error

We analyze finite-size effects and Trotter error for S and S_{Kelvin} in Supplementary Fig. 7.

Taking $U/t = 6$ and $t'/t = -0.25$ as an example, differences between results obtained with 8×8 and 12×12 clusters are minimal for S_{Kelvin} in Supplementary Fig. 7a, and are the same order of magnitude as the statistical errors for S in Supplementary Fig. 7d. The extent of finite-size effects changes with t'. For $U/t = 6$ and $t'/t = 0$ in Supplementary Fig. 7b, small finite-size discrepancies between S_{Kelvin} obtained with 8×8 and 12×12 clusters can be observed at high doping. However, these differences do not impact the overall doping dependence. Moreover, further increasing the lattice size to 16×16 shows minimal difference compared to the 12×12 lattice. In Supplementary Fig. 7e, differences between S obtained with 8×8 and 12×12 clusters are the same order of magnitude as the statistical errors. Higher doping, smaller U, and lower temperature generally causes larger finite-size effects, as the system becomes more delocalized. Therefore, our analysis up to 30% doping, with $U/t = 6$, including both $t'/t = -0.25$ and $t'/t = 0$, and down to the lowest accessible temperatures provides an approximate upper limit for finite-size effects, given the parameters considered in this work.

For two sets of parameters, $U/t = 6$, $t'/t = -0.25$ and $U/t = 10$, $t'/t = 0$, differences between results obtained with $d\tau = 0.05/t$ and $d\tau = 0.025/t$ are minimal for S_{Kelvin} in Supplementary Fig. 7a and 7c, and are the same order of magnitude as the statistical errors of S in Supplementary Fig. 7d and 7f. Larger U generally causes larger Trotter error, so our analysis up to $U/t = 10$ provides an approximate upper limit for Trotter error for data presented in the main text of this work.

[1] B. Efron and R. Tibshirani, *An Introduction to the Bootstrap* (Chapman & Hall/CRC, 1993).

[2] J. W. Tukey, Bias and confidence in not-quite large samples, Ann. Math. Statist. **29**, 614 (1958).

[3] W. O. Wang, J. K. Ding, Y. Schattner, E. W. Huang, B. Moritz, and T. P. Devereaux, The Wiedemann-Franz law in doped Mott insulators without quasiparticles, arXiv:2208.09144 (2022).

[4] E. W. Huang, R. Sheppard, B. Moritz, and T. P. Devereaux, Strange metallicity in the doped Hubbard model, Science **366**, 987 (2019).

[5] W. O. Wang, J. K. Ding, B. Moritz, E. W. Huang, and T. P. Devereaux, Magnon heat transport in a two-dimensional Mott insulator, Phys. Rev. B **105**, L161103 (2022).

[6] D. Bergeron and A.-M. S. Tremblay, Algorithms for optimized maximum entropy and diagnostic tools for analytic continuation, Phys. Rev. E **94**, 023303 (2016).

[7] B. S. Shastry, Electrothermal transport coefficients at finite frequencies, Rep. Prog. Phys. **72**, 016501 (2008).

[8] A. Bulusu and D. Walker, Review of electronic transport models for thermoelectric materials, Superlattices Microstruct. **44**, 1 (2008).

[9] A. Reymbaut, A.-M. Gagnon, D. Bergeron, and A.-M. S. Tremblay, Maximum entropy analytic continuation for frequency-dependent transport coefficients with nonpositive spectral weight, Phys. Rev. B **95**, 121104 (2017).

[10] G. D. Mahan, *Many-particle physics* (Springer New York, NY, 2000).

[11] M. R. Peterson and B. S. Shastry, Kelvin formula for thermopower, Phys. Rev. B **82**, 195105 (2010).

[12] S. Mukerjee and J. E. Moore, Doping dependence of thermopower and thermoelectricity in strongly correlated materials, Appl. Phys. Lett. **90**, 112107 (2007).

[13] S. Mukerjee, Thermopower of the Hubbard model: Effects of multiple orbitals and magnetic fields in the atomic limit, Phys. Rev. B **72**, 195109 (2005).

[14] G. Beni, Thermoelectric power of the narrow-band Hubbard chain at arbitrary electron density: Atomic limit, Phys. Rev. B **10**, 2186 (1974).

[15] C. H. Mousatov, I. Esterlis, and S. A. Hartnoll, Bad metallic transport in a modified Hubbard model, Phys. Rev. Lett. **122**, 186601 (2019).

[16] P. M. Chaikin and G. Beni, Thermopower in the correlated hopping regime, Phys. Rev. B **13**, 647 (1976).

[17] P. Phillips, T.-P. Choy, and R. G. Leigh, Mottness in high-temperature copper-oxide superconductors, Rep. Prog. Phys. **72**, 036501 (2009).

[18] S. Chakraborty, D. Galanakis, and P. Phillips, Emergence of particle-hole symmetry near optimal doping in high-temperature copper oxide superconductors, Phys. Rev. B **82**, 214503 (2010).
